# Disparities in Diabetes in Pregnancy and the Role of Social Determinants of Health

**DOI:** 10.1007/s11892-025-01587-1

**Published:** 2025-05-14

**Authors:** Laura T. Dickens

**Affiliations:** Section of Adult and Pediatric Endocrinology, Diabetes, and Metabolism, University of, Chicago, 5841 S. Maryland Ave, MC 1027, Chicago, IL 60637 USA

**Keywords:** Diabetes, Gestational diabetes, Pregnancy, Healthcare disparities, Social determinants of health

## Abstract

**Purpose of Review:**

The rates of diabetes in pregnancy (type 1, type 2, and gestational diabetes) are increasing. Diabetes in pregnancy is associated with increased risk for maternal and neonatal complications. Certain groups are disproportionately affected by these complications and this paper reviews the data about disparities in diabetes in pregnancy and explores the social determinants of health (SDoH) underlying these disparities.

**Recent Findings:**

Rates of diagnosis of gestational diabetes and pregestational diabetes are higher in racial and ethnic minority groups and people with socioeconomic disadvantage. There is higher all cause maternal mortality for Black people compared to White people. Emerging data suggests higher risk for adverse pregnancy outcomes for Black, American Indian, and Hispanic/Latina subjects with diabetes compared to White subjects. Individuals living in neighborhoods with higher poverty and less educational attainment also have higher rates of pregnancy and neonatal complications with diabetes.

**Summary:**

Diabetes in pregnancy is a complex condition which requires specialty care that can be time-consuming and costly. Individuals with disadvantages in income and employment, food security, social protection and support, and access to affordable and quality health services may be particularly susceptible to adverse outcomes of diabetes in pregnancy. Providers can reduce disparities by recognizing individuals with vulnerabilities in SDoH and tailoring treatment to social context. Equitable access to diabetes technology and postpartum care can also reduce disparities in outcomes.

## Introduction

Diabetes has been described as a “silent epidemic” which is projected to affect 1 in 8 adults (783 million people) by the year 2045 and is one of the top 10 causes of death worldwide [1–3]. Diabetes in pregnancy is of particular concern due to its high prevalence and the short- and long-term implications for maternal and offspring health. In general, diabetes in pregnancy is divided into pregestational diabetes (type 1 and type 2) and gestational diabetes (GDM), which is glucose intolerance that develops during pregnancy. In the US, GDM affected 8.3% of pregnancies in 2021, an increase from 6.0% of pregnancies in 2016 [4]. Pre-gestational diabetes affects 1–2% of pregnancies and from 2000 to 2019 the prevalence of type 2 diabetes (T2D) in pregnancy quadrupled [5]. These statistics are not surprising; amongst women of childbearing age, 4.5% have diabetes and up to 30% of those are unaware of their diagnosis [6].

The potential for adverse outcomes from diabetes in pregnancy depends on the timing and severity of hyperglycemia. With diabetes in pregnancy, there is maternal risk for pre-eclampsia and gestational hypertension and neonatal risk for stillbirth, major congenital anomaly, and large for gestational age [7, 8]. The implications of diabetes in pregnancy extend beyond pregnancy and delivery. People with gestational diabetes have up to a 50% increased lifetime risk of type 2 diabetes depending on the length of follow up and population studied [9]. The risk for type 2 diabetes after GDM remains elevated for more than 35 years after pregnancy [10]. Children of pregnancies with gestational diabetes have increased risk for overweight and obesity as well as T2D and hypertension [11, 12]. Certain groups are disproportionately affected by diabetes in pregnancy and the associated complications. This paper reviews the data about disparities in diabetes in pregnancy and explores the social determinants of health (SDoH) underlying these disparities.

Healthcare disparities can be defined as a “difference between population groups in the way they access, experience, and receive healthcare [13].” The World Health Organization, Centers for Disease Control, and many other public health groups have recently focused efforts to understand social determinants of health (SDoH) which can contribute healthcare disparities [14, 15]. Research in disparities commonly reports on race, ethnicity, and poverty because these measures are relatively straightforward to quantify from public records and existing databases. However, there are less easily quantifiable factors which are important to consider such as social and community context and healthcare access and quality. It is important to acknowledge the limitations of descriptions of individuals based on race and ethnicity. Rather than describing biologic differences, race is a social construct and its use in research and clinical medicine can have potentially deleterious effects on health outcomes and social determinants of health [16]. This paper will discuss outcomes based on race and ethnicity as reported in the cited studies while recognizing these limitations.

## Disparities in Diabetes in Pregnancy

### Overall Diabetes Prevalence and Outcomes

The prevalence of diabetes in the US varies significantly based on race and ethnicity and socioeconomic status. According to CDC data from 2019 to 2021, the prevalence of diagnosed diabetes is 13.6% for American Indian and Alaska Native adults, 12.1% for non-Hispanic Black adults, 11.7% for Hispanic adults, 9.1% for non-Hispanic Asian adults, and 6.9% for non-Hispanic White adults [17]. Prevalence of diabetes is higher for adults with less than a high school education (13.1%) compared to those with a high school education (9.1%) or more than a high school education (6.9%) [17]. The odds of having diabetes are higher for Black and White individuals living in poor neighborhoods, defined as neighborhoods with > = 20% of families below the federal poverty line [18].

Outcomes of diabetes also differ based on race and ethnicity and socioeconomic status. Zakaria et al. in 2023 observed higher odds of poor glycemic control (hemoglobin A1c 7.0% or greater) in Hispanic or Latino (OR 1.46) and non-Hispanic Black people (OR 1.28) compared to non-Hispanic White people with a self-reported diagnosis of diabetes [19]. Mortality from diabetes is higher in Black people compared to White people, as demonstrated in a recent analysis of cause-specific mortality rates across racial-ethnic groups from 2000 to 2019. This study observed that that Black people with diabetes and kidney disease had higher mortality rates compared to white people in 99.1% of 1486 US counties [20]. For type 1 diabetes, low socioeconomic status has been associated with higher morbidity and mortality and specifically with higher risk for diabetic ketoacidosis [21, 22].

### Diabetes in Pregnancy: Prevalence

Regarding diabetes in pregnancy, data has consistently demonstrated disparities in rates of diagnosis of GDM and pregestational diabetes based on race and ethnicity. A study by Shah et al. in 2021 examined data for 12,610,235 subjects in the US with singleton first live births from 2011 to 2019. Rates of GDM increased in all racial and ethnic subgroups and certain subgroups were disproportionately affected. Most Asian and Hispanic/Latina subgroups had higher rates of GDM compared to non-Hispanic white subjects, though the rates for Black subjects were similar to White subjects (risk ratio 0.97 with 95% CI 0.94–0.99). The highest absolute rates of GDM were observed in Asian Indian subjects (129.1 per 1000 live births). Higher rates of pregestational diabetes per 1000 live births were observed in Black subjects (12.4) and Hispanic/Latina subjects (10.8) compared to non-Hispanic White subjects (7.9) [23].

In addition to race and ethnicity, community factors can influence rates of GDM and pregestational diabetes. A multicenter study by Field et al. published in 2024 examined 9155 nulliparous subjects at eight US medical centers and used the Area Deprivation Index to quantify socioeconomic disadvantage. Subjects living in the highest tertile of socioeconomic disadvantage had higher rates of pregestational diabetes compared to the lowest tertile (2.6% vs. 0.8%; adjusted odds ratio, 2.52; 95% CI 1.41–4.48). No significant associations between socioeconomic disadvantage and rates of GDM were observed. However, small increases in risk for gestational diabetes were seen in subjects living in food deserts and less walkable neighborhoods [24]. Individuals in rural areas are also more likely to be diagnosed with pregestational and GDM compared to those in urban areas [25].

### Diabetes in Pregnancy: Outcomes

Though many studies have investigated racial and ethnic differences in diabetes diagnosis in pregnancy, there is relatively less data about differences in pregnancy outcomes in different population groups. Studies have consistently observed higher all cause maternal mortality rates for non-Hispanic Black people. A 2021 study of US vital records from 2016 to 2017 demonstrated 3.55 times higher maternal mortality rate for non-Hispanic Black people compared to non-Hispanic White people. The cause of mortality was predominantly cardiovascular conditions including pre-eclampsia and postpartum cardiomyopathy. Diabetes was not reported in this dataset, but these cardiovascular conditions have a clear link to diabetes [26].

A cohort study of 7,468 subjects with GDM at Kaiser Permanente Northern California observed a higher prevalence of LGA in Black subjects (25.1%) compared to Hispanic (17.3%), white (16.4%), or Asian (13.9%) individuals. A multivariate model adjusting for racial and ethnic group, parity, age at delivery, education, BMI and other variables found Black subjects were 30% more likely to have an LGA newborn [27]. Another cohort study of 32,193 pregnancies with GDM observed higher odds of multiple complications in Black subjects including preeclampsia (aOR = 1.57) neonatal hypoglycemia (aOR = 1.79), and preterm delivery < 37 weeks (aOR = 1.56) [28].

A more recent 2022 study by Venkatesh et al. described 1,560,822 subjects with gestational diabetes and singleton live births between 2014 and 2020. Black subjects had significantly increased risk for most adverse pregnancy outcomes compared to White subjects including cesarean delivery, preeclampsia or gestational hypertension, preterm birth, and NICU admission. American Indian subjects had significantly increased risk for most adverse pregnancy outcomes and Hispanic/Latina subjects also had increased risk for cesarean delivery, preterm birth, and NICU admission [8].

A 2024 study of 2,071 pregnancies with GDM examined neighborhood factors and risk for a composite outcome of complications including preeclampsia, macrosomia, hypoglycemia, neonatal intensive care unit admission, hemorrhage, and stillbirth. They observed higher rates of complications in Black subjects compared to White or other (37.8% vs. 31.0% and 26.9%, *p* = 0.0009). Complications were more likely in neighborhoods with > 5.7% of households below the poverty level and in neighborhoods with > 8.9% of residents with less than a high school diploma [29].

### Understanding the Reason for Disparities in Diabetes in Pregnancy

The data presented above establishes that rates of diabetes in pregnancy and pregnancy complications disproportionately affect certain population groups. Treatment of diabetes in pregnancy with lifestyle measures and pharmacologic therapy to improve glycemia reduces risk for maternal and neonatal complications for GDM and pregestational diabetes [30, 31]. However, the care required to optimize outcomes for diabetes in pregnancy can be complex, time-consuming, and costly. We will explore the requirements of diabetes care in pregnancy to understand factors that can limit certain groups of people from receiving optimal care.

Diabetes care in pregnancy can be complex. Qualitative studies of women with GDM have demonstrated limited patient understanding about different types of diabetes, the effect of diabetes on pregnancy outcomes, and the long term health implications [32]. Registered dieticians and certified diabetes educators provide critical counseling about nutrition and diabetes self-management with additional support from patient navigators and community health workers. However, these resources may not be universally available. Medical care for diabetes in pregnancy requires specific provider knowledge and technologic expertise. The American Diabetes Association (ADA) and Society for Maternal and Fetal Medicine (SMFM) provide lengthy recommendations and checklists for management of diabetes from pre-conception through antenatal and postpartum care [33, 34]. Limited access to high quality medical centers and subspecialists is a factor that can contribute to disparities in diabetes in pregnancy.

Diabetes care in pregnancy can be time-consuming. The foundation of management of diabetes in pregnancy is lifestyle measures including nutrition and physical activity [33]. It is essential for patients to have time to shop for food and prepare meals and time for physical activity. Furthermore, significant time is required for glycemic monitoring and medication administration. Glycemic monitoring in pregnancy is often recommended fasting, preprandial, and postprandial (6 times per day) and medical therapy with insulin can require injections up to 4–5 times per day [33]. Frequent medical visits are required to monitor glycemia and fetal wellbeing. These can include provider appointments (obstetrics or maternal fetal medicine, endocrinology, nutritionist) and dedicated ultrasounds. By the end of pregnancy, twice weekly antenatal testing is recommended in pregnancies with pregestational diabetes [35]. Attending to glycemic monitoring, medication administration, and frequent medical appointments can be challenging for patients with work and childcare obligations especially when there is limited social support.

Diabetes care in pregnancy can be costly. The interventions described above including healthy foods, medications, testing supplies, and transportation to medical visits can lead to significant cumulative costs for diabetes in pregnancy. As an example, continuous glucose monitors (CGM) have become the standard of care for type 1 diabetes outside of pregnancy and show evidence of benefit in pregnancy for pregestational diabetes and possibly gestational diabetes [36–38]. CGM devices are worn on the body for 7–15 days and measure interstitial glucose to provide continuous glucose data and obviate the need for fingersticks. This technology has potential to ease the burden of the intensive fingerstick glucose testing that is required in pregnancy but access to CGM can be limited due to cost and insurance coverage. According to 2021 data from the Centers for Disease control (CDC), Medicaid provided coverage for 41.0% of deliveries overall and specifically 64.0% of Black patients at 58.1% of Hispanic patients. Medicaid for CGM coverage varies by state and as of 2022, 40 states and the District of Columbia provided some level of CGM coverage but with varying requirements for diagnosis and documentation [39].

Insulin cost can pose another significant financial burden. Most Medicaid plans cover insulin for free or significantly reduced cost though this too can vary by state [40]. With private insurance, many states have enacted copay caps for insulin cost ranging from $25-$100 per month. However, these out of pocket costs may still be prohibitive for low income patients, particularly considering cost of testing supplies in addition to medications.

Financial strain around the time of pregnancy has been well documented and disproportionately affects Black and Hispanic individuals. According to a 2023 study of US national survey data, poverty rates among Black pregnant subjects before birth was 47.3% and in the month after birth was 54.6%. Among Hispanic pregnant subjects the poverty rate before birth was 36.3% and in the month after birth was 49.7% [41]. There are myriad factors contributing to the baseline poverty rates and increase in poverty after birth. One important contributing factor to financial strain is parental leave. Black and Hispanic workers are less likely than White workers to have access to and use paid parental leave though these effects are somewhat attenuated by employment characteristics (full or part time work, occupation, type of industry) [42].

**Fig. 1:**
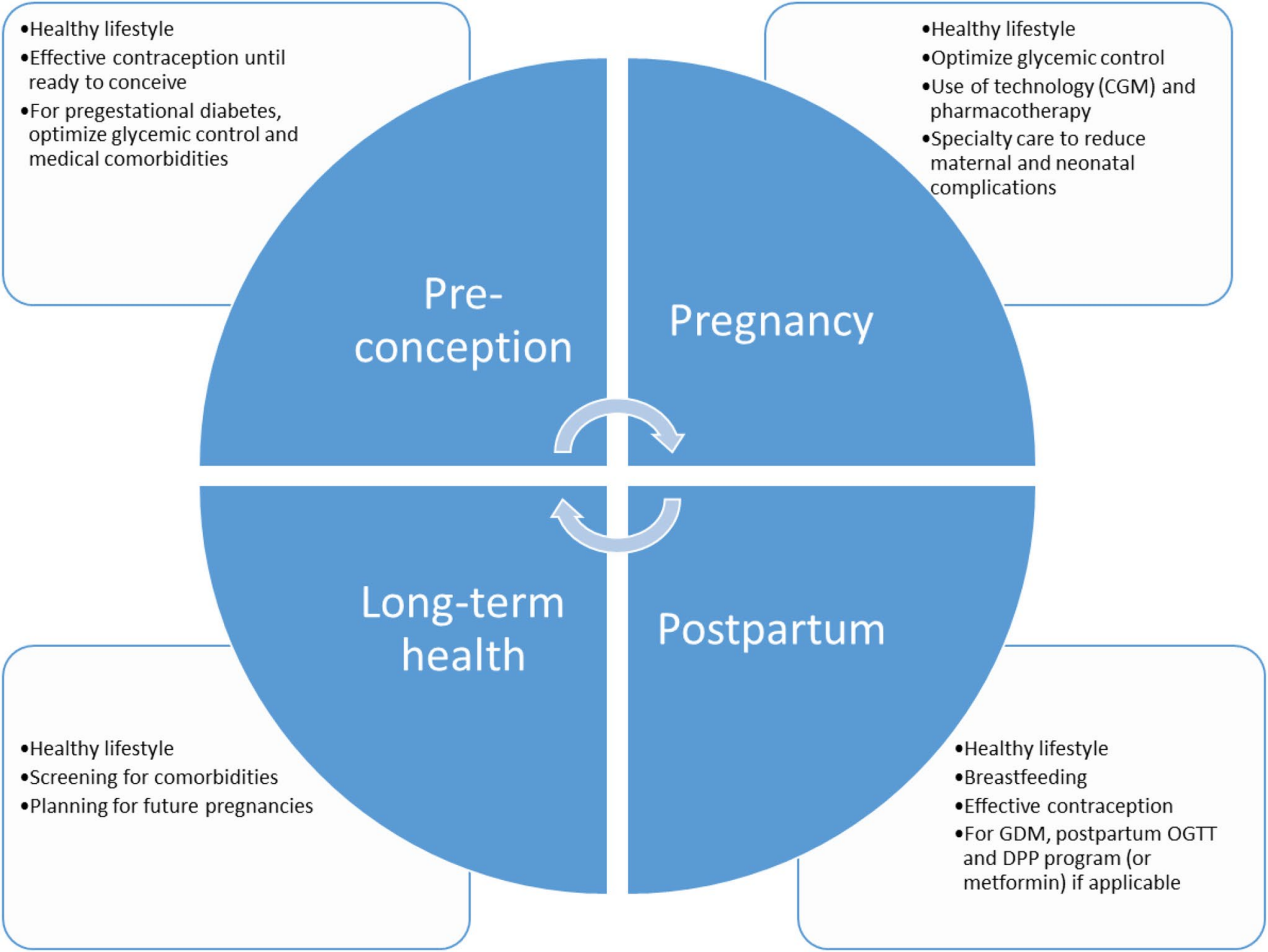
The continuum of diabetes care before, during, and after pregenency

Diabetes care does not end with delivery. Figure 1 illustrates the continuum of diabetes care through pregnancy, postpartum, and long-term health. A particularly critical step for long-term health in people with GDM is detecting postpartum dysglycemia. It has been well established that intensive lifestyle interventions through Diabetes Prevention Programs and use of metformin can reduce risk for type 2 diabetes by around 50% for people with a history of GDM and impaired glucose tolerance. However, many individuals do not complete postpartum testing to identify impaired glucose tolerance and are therefore unable to benefit from these interventions. Historically < 50% of people complete the postpartum OGTT due to barriers in transportation, finances, and childcare [43].

Income and employment, food security, social protection and support, and access to affordable and quality health services are SDoH which can contribute significantly to disparities in diabetes in pregnancy. Pregnant patients with vulnerabilities in any of these SDoH may have difficulty accessing care for diabetes in pregnancy and adhering to the time-consuming and costly treatments that are required.

### Strategies for Reducing Disparities and Improving Outcomes

In order to address health inequities related to SDoH on an individual patient level, healthcare providers must recognize when vulnerabilities exist. The National Academy of Sciences created a framework for educating health professionals to address SDoH and health inequalities [44]. This should be a critical component of education for new providers and continuing education for current providers. The ADA Standards of Care in 2024 includes recommendations for tailoring diabetes treatment for social context. These recommendations advise providers to assess food and housing insecurity, financial barriers, and social community support to guide treatment decisions. Diabetes care providers and clinics should have a structured approach to identify barriers to care and health systems should have programs in place to provide assistance. A 2024 study of 68 hospitals in Michigan found that systematic screening for SDoH was performed in less than half of the surveyed hospitals but did appear to be feasible in different types of hospitals [45].

The ADA additionally recommends involvement of local community resources including health coaches, navigators, or community health workers (CHW) [46]. One example of a successful community health worker initiative is the Safe Start program in Philadelphia. The program enrolled publicly insured pregnant women with chronic health conditions including diabetes and paired the subjects with a CHW to provide case management, care coordination, and emotional support. Compared to a control group with usual care, the Safe Start participants had more engagement in care, fewer antenatal admissions, and short neonatal intensive care unit (NICU) stays [47].

Optimized single center care for diabetes in pregnancy could reduce the risk for adverse outcomes in pregnancy in vulnerable populations. A 2023 study from a socially deprived suburb in Paris observed no difference in pregnancy outcomes for subjects with hyperglycemia with and without psychosocial deprivation or food insecurity. This center utilizes a multidisciplinary team including a diabetologist, obstetrician, midwife, dietician, and nurse educator with close monitoring and a tailored education program [48]. Telehealth may reduce the burden of in person healthcare visits for people with diabetes in pregnancy but data for improved outcomes is lacking [49].

Immediate postpartum oral glucose tolerance test (OGTT) is a promising alternative to standard 4–12 week postpartum screening with the potential to improve access to care. A 2020 study by Werner et al. recruited 300 subjects to complete a 2 day postpartum OGTT followed by a standard 4–12 week postpartum OGTT and then hemoglobin A1c one year postpartum. In this cohort, there was no significant difference between the immediate postpartum and standard OGTT for predicting dysglycemia (A1c > 5.7%) at one year postpartum. Completion of the 2 day OGTT was nearly universal (99% of subjects) [50]. Recent American College of Obstetricians and Gynecologists (ACOG) guidelines presents immediate postpartum 75 g OGTT as a reasonable alternative to the standard 4–12 week postpartum screening [51].

Health care policy can takes steps to mitigate disparities, particularly relating to equitable access to diabetes technology. Universal insurance coverage for CGM for patients using insulin can improve glycemic control, enhance patient safety and ease the burden of frequent fingersticks. An initiative from the Center for Health Care Strategies (CHCS) called Accelerating Access to CGMs in Medicaid to Improve Diabetes Care is working to help states improve access to CGM for people with Medicaid [52]. Furthermore, universal insurance coverage for postpartum care in the “fourth trimester” (birth to 12 weeks postpartum) can help retain patients in ongoing care, especially those at high risk for future diabetes and diabetes complications.

## Conclusions

Recognizing that health disparities exist is the first critical step in reducing inequities and optimizing health of the entire population. This paper has outlined the current knowledge about disparities in diabetes in pregnancy and the factors underlying these disparities. Further study is needed to expand the relatively limited data about the impact of disparities on pregnancy outcomes. Diabetes care providers should be knowledgeable about the impact of SDoH on pregnancies with diabetes and take proactive steps to connect patients with healthcare and community resources. Improving access to diabetes medications and technology is another critical approach to reduce disparities and diabetes care providers can be important advocates in these efforts.

## Key References

* Shah NS, Wang MC, Freaney PM, et al. (2021) Trends in Gestational Diabetes at First Live Birth by Race and Ethnicity in the US, 2011–2019. JAMA 326:660–669.

A cross-sectional study of 12,610,235 subjects with singleton live births which showed increasing rates of GDM in all racial and ethnic subgroups from 2011 to 2019 and that most Asian and Hispanic/Latina subgroups had higher rates of GDM compared to non-Hispanic white people

** Field C, Grobman WA, Yee LM, et al. (2024) Community-level social determinants of health and pregestational and gestational diabetes. Am J Obstet Gynecol MFM 6:101249.

A study of 9155 nulliparous subjects which showed that subjects living in the highest tertile of socioeconomic disadvantage had higher rates of pregestational diabetes compared to the lowest tertile.

** Venkatesh KK, Lynch CD, Powe CE, Costantine MM, Thung SF, Gabbe SG, Grobman WA, Landon MB (2022) Risk of Adverse Pregnancy Outcomes Among Pregnant Individuals With Gestational Diabetes by Race and Ethnicity in the United States, 2014–2020. JAMA 327:1356–1367.

A cross-sectional study of 1,560,822 subjects with GDM which showed higher rates of adverse pregnancy outcomes in Black, American Indian, and Hispanic/Latina subjects compared to White subjects.

**Thomas LV, Jurkovitz CT, Zhang Z, Fawcett MR, Lenhard MJ (2024) Neighborhood Environment and Poor Maternal Glycemic Control-Associated Complications of Gestational Diabetes Mellitus. AJPM Focus 3:100201.

A study of 2,071 pregnancies with GDM which showed higher rates of pregnancy complications in Black subjects compared to White subjects and in individuals living in neighborhoods with more poverty and less educational attainment.

## Data Availability

No datasets were generated or analysed during the current study.
